# *Chlorella vulgaris* Attenuates Dermatophagoides Farinae-Induced Atopic Dermatitis-Like Symptoms in NC/Nga Mice

**DOI:** 10.3390/ijms160921021

**Published:** 2015-09-02

**Authors:** Heerim Kang, Chang Hyung Lee, Jong Rhan Kim, Jung Yeon Kwon, Sang Gwon Seo, Jae Gab Han, Byung Gon Kim, Jong-Eun Kim, Ki Won Lee

**Affiliations:** 1 WCU Biomodulation Major, Department of Agricultural Biotechnology, Seoul National University, Seoul 151-921, Korea; E-Mails: heeerim@gmail.com (H.K.); changhyungds@naver.com (C.H.L.); allmendhubel@gmail.com (J.R.K.); seo0414@naver.com (S.G.S.); 2 Advanced Institutes of Convergence Technology, Seoul National University, Suwon 443-270, Korea; E-Mail: jungykwon@snu.ac.kr; 3 Department of Biomaterials Science and Engineering, Yonsei University, Seoul 120-749, Korea; E-Mail: jaegab@daesang.com; 4 Department of Health Food Research & Development of Daesang Corp., Icheon 467-813, Korea; E-Mail: gonee@daesang.com; 5 Interdisplinary course of Science for Aging, Graduate School, Yonsei University, Seoul 120-749, Korea; 6 Research Institute of Bio-Food Industry, Institute of Green Bio-Science and Technology, Seoul National University, Pyeongchang 232-916, Korea

**Keywords:** atopic dermatitis, chlorella vulgaris, immune cell infiltration, inflammatory cytokines, inflammatory skin lesions

## Abstract

Atopic dermatitis (AD) is a chronic and inflammatory skin disease that can place a significant burden on quality of life for patients. AD most frequently appears under the age of six and although its prevalence is increasing worldwide, therapeutic treatment options are limited. *Chlorella vulgaris* (CV) is a species of the freshwater green algae genus chlorella, and has been reported to modulate allergy-inducible factors when ingested. Here, we examined the effect of CV supplementation on AD-like symptoms in NC/Nga mice. CV was orally administrated for six weeks while AD-like symptoms were induced via topical application of *Dermatophagoides farinae* extract (DFE). CV treatment reduced dermatitis scores, epidermal thickness, and skin hydration. Histological analysis also revealed that CV treatment reduced DFE-induced eosinophil and mast cell infiltration into the skin, while analysis of serum chemokine levels indicated that CV treatment downregulated thymus- and activation-regulated chemokine (TARC) and macrophage-derived chemokine (MDC) levels. In addition, CV treatment downregulated mRNA expression levels of IL-4 and IFN-γ. Taken together, these results suggest that CV extract may have potential as a nutraceutical ingredient for the prevention of AD.

## 1. Introduction

Atopic dermatitis (AD) is a common chronic skin immune disorder that triggers continuous itching [1]. Over the last decade, the prevalence of AD has increased by over 20% in children, attributable to changes in lifestyles and the surrounding environment. Approximately 60% of AD patients will display symptoms before the age of six [2,3]. AD is commonly considered as the first step of the “atopic march”, a typical progression of allergic diseases which frequently first occurs during childhood [4]. A recent cohort study revealed that AD patients had nearly a three-fold increased risk of developing rhinitis and asthma [5].

AD can be caused by genetic mutations, skin barrier damage, and imbalances of innate immune cell activity, such as those elicited by Th1 and Th2 cells [6]. During the development of AD, the skin structure collapses due to continuous itching and inflammation, leading to elevated transepidermal water loss (TEWL). TARC (thymus- and activation-regulated chemokine, CCL17) and MDC (macrophage-derived chemokine, CCL22) are two Th2 cell-producing chemokines that are involved in the pathogenesis of AD and their expression levels are known to be significantly higher in AD patients when compared to normal subjects [7]. House dust mites promote the activation and maturation of dendritic cells that go on to induce Th2-cell differentiation and upregulate TARC and MDC levels [8,9]. These chemokines recruit Th2 lymphocytes to inflammatory sites and aggravate allergic reactions. IL-4, a Th2 cell-induced cytokine, induces AD pathogenesis together with Th2-type responses [10]. Other Th1 cell-derived cytokines such as IFN-γ induce apoptosis in keratinocytes, leading to the development of AD skin lesions [11]. Hyperplasia and dense infiltration of eosinophils and mast cells are also detected in the skin layers of mouse model with AD [12,13]. Despite the numerous mechanistic and clinical studies that have been conducted, the effective treatment and management of AD remains challenging. In addition, the adverse effects of current treatments such as telangiectasia on the cheeks, bacterial infection, acne, and hypertrichosis are often evident and these effects can result in some degree of steroid phobia for many patients [14]. It is also known that corticosteroid addiction syndrome can cause both local and systemic side effects [15]. Therefore, the prevention and treatment of AD using natural, safe, and effective natural compounds is desirable.

Chlorella is a genus of single-celled green algae which contains amino acids, protein, vitamins, dietary fiber, and a variety of antioxidants, bioactive materials, and chlorophylls [16]. It is considered a potential food source due to its high protein content and other nutritional components. Particularly in East Asian countries such as Japan, Taiwan, and Korea, chlorella is commonly consumed with rice, tea, and pancakes [17]. Through various studies, chlorella has been reported to exhibit antitumor, anti-obesity, and hypoglycemic effects in diabetic mice [18,19,20]. Interestingly, one species of chlorella, *Chlorella vulgaris* (CV), contains high concentrations of protein (51%–58% of dry matter) as well as a host of other biological and disease-preventive functions [21]. CV consumption may also have protective effects in cases of acute hepatic damage and may induce antioxidant activities [22]. In terms of immune-modulating effects, CV supplementation inhibits the production of immunoglobulin E (IgE) and enhances the Th1 cell-related immune response [23]. In addition, it has been reported that oral administration of CV elicits inhibitory effects by suppressing histamine secretion in clinical studies [24]. However, a definitive correlation between CV consumption and allergic diseases such as atopic dermatitis remains unclear. We sought to investigate whether a chlorella-enriched diet may have suppressive effects on the development of AD-like skin symptoms.

## 2. Results

### 2.1. CV Supplementation Alleviates DFE-Induced AD-Like Skin Lesions in NC/Nga Mice

**Figure 1:**
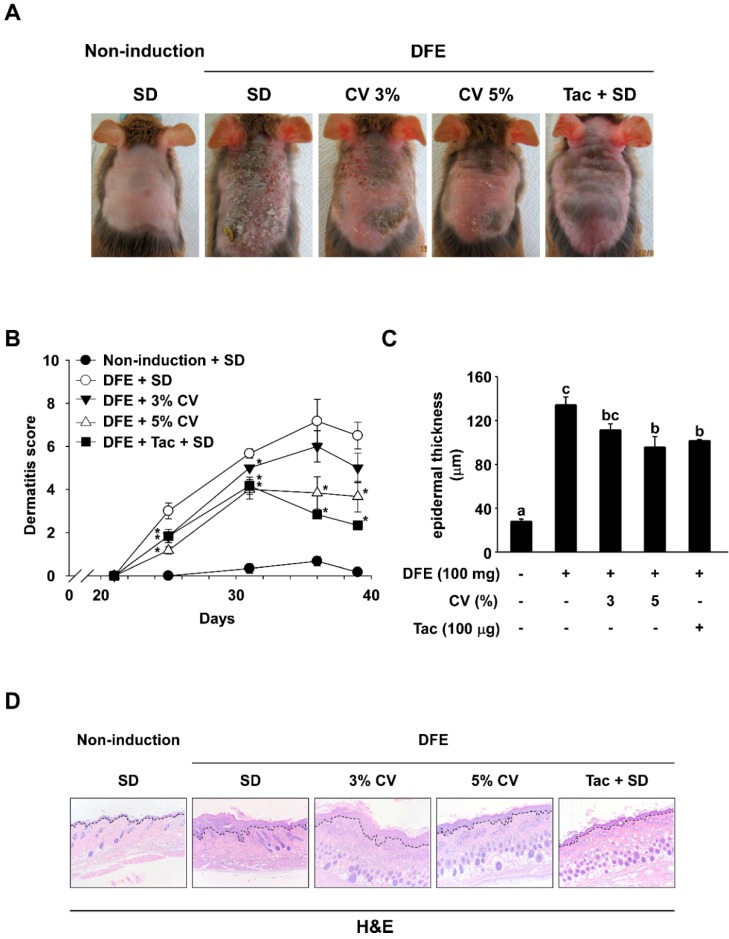
Effect of CV on DFE-induced AD-like symptoms in NC/Nga mice. **(A)** Skin lesions typical of AD. Images of skin lesions were taken in the sixth week; (**B**) Dermatitis scores. The scoring represents a clinical index that was evaluated each week from Day 21 to 39; (**C**) Epidermal thicknesses of skin lesions were measured until the final week; (**D**) H&E stained skin recovered in the sixth week (100×). Dotted lines indicate epidermal hypertrophy. Data represent mean values ± SEM (*n* = 6). Mean values with letters (a–c) within a graph are significantly different from each other at *p* < 0.05. (* *p* < 0.05, Student’s *t*-test). CV: *Chlorella vulgaris*; DFE: *Dermatophagoides farinae* extract; AD: atopic dermatitis; H&E: hematoxylin and eosin; Tac: tacrolimus.

To investigate the effect of CV intake on DFE-induced AD symptoms, images of dorsal skin were used to evaluate dermatitis scores and measure epidermal thicknesses. In the final week, AD-like symptoms such as erythema, edema, excursion, and dryness were observed. However, animals supplemented with CV or topical application of tacrolimus exhibited dramatic reductions in AD-like skin lesions (Figure 1A). Supplementation of 3% or 5% CV (5.00 ± 0.68 and 3.67 ± 0.71) significantly lowered dermatitis scores in comparison to the standard diet (6.50 ± 0.62) (Figure 1B). Dorsal skin thicknesses of NC/Nga mice were also increased in the DFE-induced groups (134.35 ± 7.12) and thickness was slightly decreased with 3% and 5% CV supplementation (111.42 ± 5.46 and 95.83 ± 9.41) (Figure 1C). The skin samples were prepared and stained with hematoxylin and eosin (H&E) and are shown in Figure 1D. AD symptom severity was observed to be decreased in the CV-supplemented group and the tacrolimus control group.

### 2.2. CV Supplementation Enhances Skin Hydration in DFE-Induced AD-Like Skin Lesions in NC/Nga Mice

**Figure 2:**
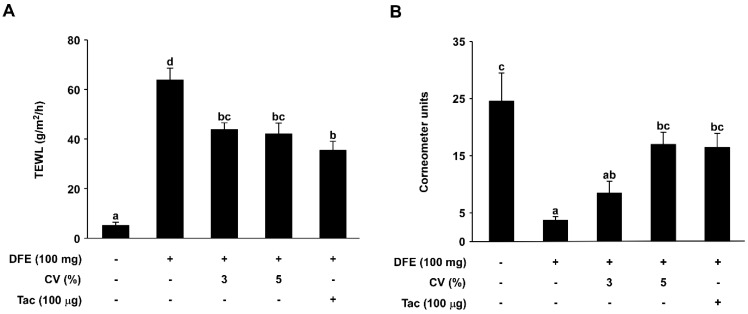
Effect of CV on DFE-induced downregulation of skin hydration. (**A**) transepidermal water loss (TEWL) in mouse dorsal skin was measured using a skin evaporative water recorder in the final week of the experiment; and (**B**) The level of skin hydration in mouse dorsal skin was also measured using a skin corneometer recorder in the final week. Data represent the mean values ± SEM (*n* = 6). Mean values with letters (a–d) within a graph are significantly different from each other at *p* < 0.05.

To further investigate the effect of CV supplementation on water loss through the skin of NC/Nga mice, total epidermal water loss was evaluated on the final day (Day 42). TEWL was significantly increased in the DFE-induced group (63.78 ± 4.81 g/m^2^/h) compared to the naïve control group (5.06 ± 1.43 g/m^2^/h). In contrast, TEWL was decreased in the 3% and 5% CV-supplemented groups (43.79 ± 2.71 and 42.00 ± 4.45 g/m^2^/h) (Figure 2A). Skin hydration in DFE-induced AD mice was also measured using a corneometer unit. The 3% and 5% CV-supplemented groups (8.48 ± 2.08 and 16.98 ± 2.16) exhibited higher skin hydration levels compared to the DFE-induced group (3.71 ± 0.64) (Figure 2B).

### 2.3. CV Supplementation Suppresses DFE-Induced Infiltration of Eosinophils and Mast Cells into Skin Lesions

**Figure 3:**
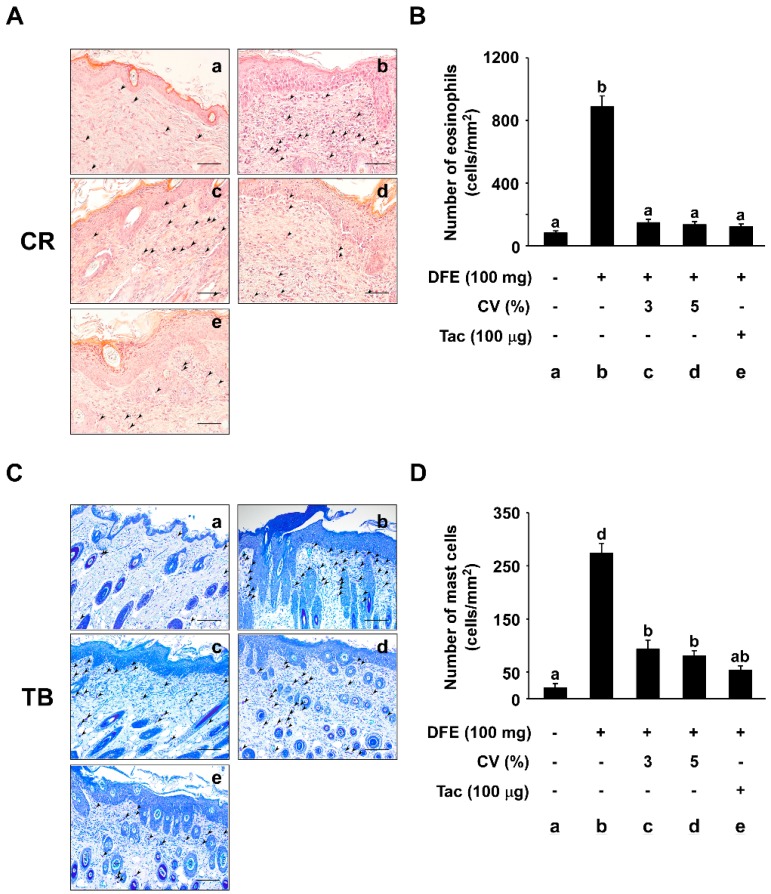
Effect of CV on DFE-induced eosinophil and mast cell infiltration into skin lesions. (**A**) Representative images depicting the histological features of skin lesions are shown. Skin lesions were stained with Congo red (CR) to identify eosinophils. The arrows indicate the CR-stained eosinophils. Eosinophils were counted under a microscope at 400× magnification. Scale bar, 50 μm; (**B**) The numbers of eosinophils in 1 mm^2^ sections of skin lesion were measured and graphed. Data represent the mean values ± SEM (*n* = 6). Mean values with letters (a–b) within a graph are significantly different from each other at *p* < 0.05; (**C**) Representative images depicting the histological features of skin lesions. Skin lesions were stained with toluidine blue (TB) to identify mast cells. The arrows indicate the TB-stained mast cells. Mast cells were counted under a microscope at 400× magnification. Scale bar, 50 μm; and (**D**) The numbers of mast cells in 1 mm^2^ sections of skin lesion were measured and graphed. Data represent the mean values ± SEM (*n* = 6). Mean values with letters (a, b, d) within a graph are significantly different from each other at *p* < 0.05.

To investigate whether CV supplementation suppresses DFE-induced infiltration of eosinophils and mast cells into skin lesions, tissue sections were stained with Congo red (CR) and toluidine blue (TB). The number of eosinophils stained with CR was significantly increased in the DFE-induced group (886.68 ± 71.12 cells/mm^2^) compared to the naïve control group (80.00 ± 14.61 cells/mm^2^) (Figure 3A). However, the number of eosinophils in the 3% and 5% CV-supplemented group (146.68 ± 22.31 and 133.33 ± 19.78 cells/mm^2^) was observed to be sharply decreased (Figure 3B). In addition, the number of TB-stained mast cells per cell/mm^2^ was quantified (Figure 3C). The number of mast cells in tissue sections was dramatically increased in the DFE-induced group (273.33 ± 19.09 cells/mm^2^) compared to that of the naïve control group (20.00 ± 8.94 cells/mm^2^) (Figure 3C), while supplementation of 3% or 5% CV caused a dose-dependent decrease in this effect (93.33 ± 16.87 and 80.00 ± 10.33 cells/mm^2^) (Figure 3D).

### 2.4. CV Supplementation Downregulates DFE-Induced Serum TARC and MDC Levels in NC/Nga Mice

**Figure 4:**
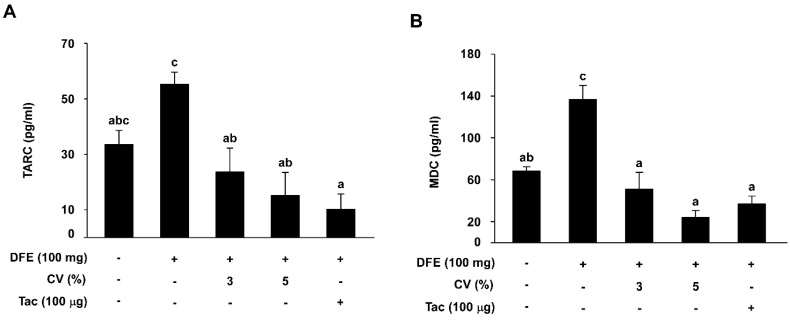
Effect of CV on DFE-induced increases in serum TARC and MDC levels in skin lesions. Levels of serum TARC (**A**, *n* = 7–8) and MDC (**B**, *n =* 7–8) were measured by ELISA. Data represent the mean values ± SEM (*n* = 7). Mean values with letters (a–c) within a graph are significantly different from each other at *p* < 0.05. TARC: thymus- and activation-regulated chemokine; MDC: macrophage-derived chemokine.

To assess TARC and MDC levels in DFE-induced NC/Nga mice, serum samples were collected on the final day of the experiment (Day 42). Serum TARC levels in the DFE-induced group (55.15 ± 4.47) were increased compared to that of the naïve group (33.45 ± 5.15). However, supplementation with 3% or 5% CV (23.60 ± 8.66 and 15.21 ± 8.26) elicited a remarkable reduction in TARC levels (Figure 4A). Measurements of serum MDC levels in the DFE-induced group (136.58 ± 13.59) were also two-fold higher than for the naive group (68.10 ± 4.27). MDC levels in the 3% (51.04 ± 16.02) and 5% CV-supplemented groups (23.98 ± 6.46) were lower than in the DFE-induced group (Figure 4B).

### 2.5. CV Supplementation Downregulates DFE-Induced Th2 and Th1 mRNA Expression Levels in NC/Nga Mice

**Figure 5:**
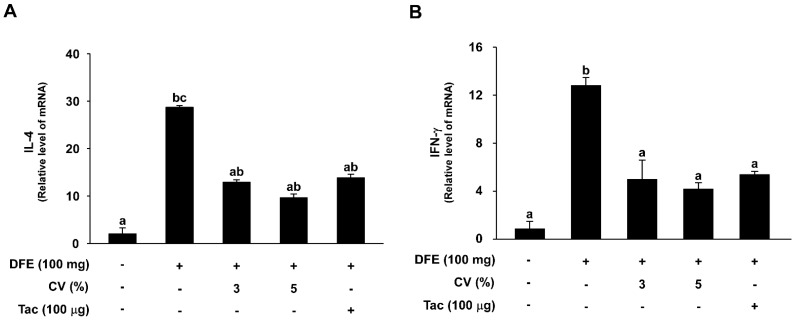
Effect of CV on DFE-induced increases in cytokine mRNA levels. IL-4 (**A**) and IFN-γ (**B**) mRNA levels were analyzed by real-time quantitative RT-PCR. Data represent the mean values ± SEM (*n* = 6). Means with letters (a–c) within a graph are significantly different from each other at *p* < 0.05.

To investigate the effect of CV supplementation on Th2 and Th1 mRNA expression levels, tissue sections were collected and analyzed. mRNA levels of IL-4, a Th2 cell-related cytokine, were significantly increased in the DFE-induced group (28.69 ± 0.40) while the control group had relatively little expression (2.03 ± 1.26). However, the 3% and 5% CV-supplemented groups, as well as the tacrolimus-treated groups, exhibited downregulated IL-4 mRNA levels (12.91 ± 0.54, 9.62 ± 0.82, and 13.81 ± 0.80) (Figure 5A). Meanwhile, mRNA levels of IFN-γ, a Th1 cell-related cytokine, were significantly elevated in the DFE-induced group (12.80 ± 0.66) compared to that of the control group (0.85 ± 0.63). Supplementation with 3% or 5% CV, as well as tacrolimus application, resulted in a sharp decrease in these levels (4.97 ± 1.62, 4.17 ± 0.53, and 5.37 ± 0.27) (Figure 5B).

## 3. Discussion

Chlorella is a promising unicellular organism for future biodiesel needs, biological materials, and as a human food source. Compared to other ordinary crop plants, chlorella grows well in a vessel or chamber, with minimal loss of water. The organism also contains a higher proportion of protein than other vegetable materials such as soybean meal and most cereals. In addition, most of the fats (75%) in chlorella are C_16_, C_18_ unsaturated fats [25]. Chlorella is therefore receiving increasing interest as a promising food source for safe, efficient, and environmentally friendly food products.

Recent clinical studies have shown that chlorella supplementation enhances natural killer cell activity and the production of Th1 cell-related cytokines in healthy subjects [26]. In addition, *in vitro* and *in vivo* studies have demonstrated that CV supplementation has an inhibitory effect on allergic immune reactions. Chlorella treatment has been shown to reduce mast cell degranulation and histamine release, while oral administration can result in a significant reduction of IgE levels in an ovalbumin-sensitized mouse model [24].

In AD patients, allergenic materials such as viral particles and pollens pass through the epidermal skin easily if the skin barrier is dysfunctional [27]. Such conditions also allow significant amounts of water to escape, leaving the skin dry [28]. Oral administration of CV has been shown to elicit positive effects for the treatment and prevention of atopic dermatitis. From a systemic point of view, many types of cytokine and chemokine are involved in the development and degenerative conditions that characterize atopic dermatitis [29]. Epithelial cells release the thymic stromal lymphopoietin cytokine after contact is made with viral particles or allergens and promote the maturation of dendritic cells, which in turn induce secretion of the chemokine TARC. TARC attracts Th2 cells toward inflammatory sites and can aggravate the symptoms of atopic dermatitis. MDC also promotes the recruitment of Th2 cells toward sites of inflammation [10]. Our results therefore suggest that CV supplementation might alleviate the symptoms of AD. The cytokine IL-4 induces B-cell class switching to IgE while enhancing production of IL-4 [30]. It also downregulates the production of Th1 cells and induces the differentiation of naive helper T cells into Th2 cells. Previous studies have demonstrated that elevated levels of IL-4 can lead to IgE synthesis in atopic dermatitis patients [31]. IFN-γ is a type 2 interferon that is important for innate and adaptive immunity. Prior studies have demonstrated that IFN-γ expression is heavily linked to the severity of atopic dermatitis [29]. We demonstrated that CV supplementation could be used to downregulate this severity by lowering IFN-γ mRNA levels.

In summary, we have shown that oral administration of CV improves atopic dermatitis-like skin lesions by modulating local and systemic immune responses. Further experiments are needed to better understand the detailed molecular mechanisms linking CV supplementation and AD severity. Clinical studies are also needed prior to its utilization by the food industry and atopic dermatitis patients. Chlorella consumption for the treatment of other allergic diseases such as allergic rhinitis and asthma may also be feasible. Additionally, the identification and isolation of compounds with anti-atopic activity from CV would provide further application possibilities. With further evidence, chlorella could become an important functional food source or health supplement for the treatment and prevention of atopic dermatitis and other allergic disorders.

## 4. Experimental Section

### 4.1. Chlorella Vulgaris (CV) Supplementation

The *Chlorella vulgaris* supplementation diet was manufactured by Daesang Co. (Seoul, Korea). First, 5 mg of CV was placed into 50 mL boiling water and stirred for 30 min. When the temperature reached 95 °C, the supernatant was collected and dried following the manufacturer’s protocol. The diet formulation was produced in pellet form. The control diet was made by mixing 2 g/kg CV or 0.005 g/kg dexamethasone (DEX) and an AIN-93G-based cereal formula (G-Bio Co., Ltd, Gwacheon, Korea).

### 4.2. Animals

Three-week-old NC/Nga male mice were purchased from SLC Japan (Tokyo, Japan). Mice were housed in individual ventilated cages under specific-pathogen-free conditions at 22 ± 2 °C with a 12 h light-dark cycle. All experimental protocols were approved by the Institutional Animal Care and Use Committee of Seoul National University, Seoul, Korea (SNU-131119-2).

**Figure 6:**
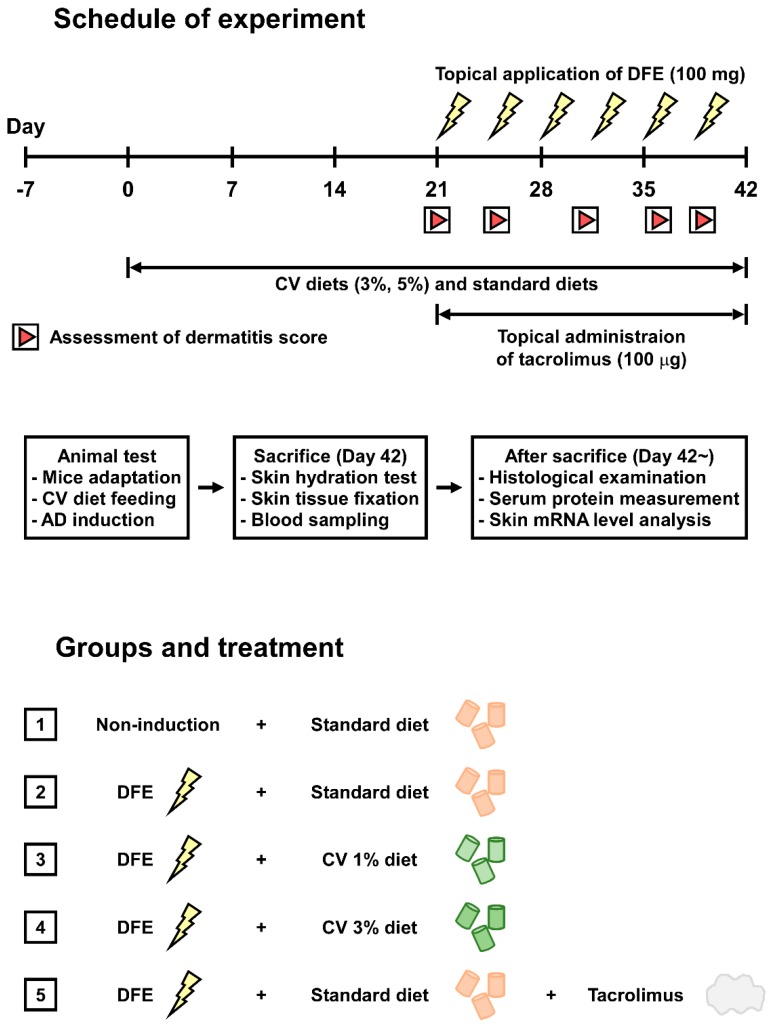
Experimental design overview. DFE: *Dermatophagoides farinae* extract; CV: *Chlorella vulgaris*; AD: atopic dermatitis.

The experimental design is depicted in Figure 6. After one week of acclimation, mice were divided into five groups (*n* = 8 per group) as follows: (1) Non-induction + standard diet; (2) *Dermatophagoide farinae* powder (DFE) + standard diet; (3) DFE + CV 3% standard diet; (4) DFE + CV 5% standard diet; and (5) DFE + tacrolimus + standard diet. Mice ingested these dietary formulae for six weeks. From the third week, the mice received topical applications of DFE (Biostir, Hiroshima, Japan) twice weekly for three weeks. Tacrolimus (0.1% Protopic^®^ Ointment, Astellas Pharma, Tokyo, Japan; 100 µg/mouse/day) was topically applied onto their shaved dorsal skin five times a week for three weeks.

### 4.3. Induction of Atopic Dermatitis (AD)-Like Symptoms

NC/Nga mice were anesthetized with 2% isoflurane (HanaPharm, Seoul, Korea) and dorsally shaved using an electric clipper (Daito Thrive, Tokyo, Japan) and shaving cream (Reckitt Benckiser, Cedex, France). To disrupt the skin barrier of the mice, 150 µL of 4% sodium dodecyl sulfate (Sigma, St. Louis, MO, USA) was topically applied. After 3 h, 100 mg Biostir-AD cream containing DFE was applied to the shaved dorsal skin, the face, and the back side of both ears of the NC/Nga mice twice weekly for three weeks to induce AD-like skin lesions.

### 4.4. Assessment of Skin Lesions and Dermatitis Scores

Mice were anesthetized with 2% isoflurane, and photographs of the skin symptoms were taken using a digital camera (Canon SX 40 HS, Tokyo, Japan) on the final week of the experiment. The dermatitis scores were evaluated on five occasions over the three weeks according to criteria described previously. Scores of 0 (none), 1 (mild), 2 (moderate), and 3 (severe) were used for each of the four symptoms (erythema/hemorrhage, edema, excoriation/erosion, and scaling/dryness). A total dermatitis score indicated the AD clinical severity and each dermatitis score was aggregated (maximum score: 12).

### 4.5. Histological Examination

To measure epidermal thickening, skin samples from each mouse were recovered on the final day of the experiment, and were fixed with 10% neutral-buffered formalin and embedded in paraffin. Then, 4-µm thick sections were cut and transferred onto slides. After deparaffinizing the skin sections and staining with hematoxylin and eosin, the tissue sections were examined at 100× magnification. To detect infiltration of eosinophils and mast cells, the skin of each mouse was prepared on the final day of the experiment, fixed with 10% neutral-buffered formalin, embedded in paraffin, sliced into sections (4 µm thick), and transferred onto slides. Deparaffinized skin sections were dyed with Congo red and toluidine blue, respectively. The number of eosinophils and mast cells per 0.025 mm^2^ skin sample was quantified at 400× magnification. Tissue sections were assessed using an Olympus AX70 light microscope (Tokyo, Japan). Epidermal thickness was examined by measuring the area-length ratio in the individual sections of mouse skin.

### 4.6. Measurement of Serum TARC and MDC Levels

Blood samples were prepared from anesthetized mice by cardiac puncture on the final day of the experiment and serum obtained from the whole blood was kept at −80 °C until further use. The total levels of TARC and MDC in the serum were examined using an enzyme-linked immunosorbent assay (ELISA) Kit (R&D Systems, Minneapolis, MN, USA), following the manufacturer’s instructions.

### 4.7. Measurement of Th2 and Th1 Cytokine mRNA Levels

To evaluate total mRNA levels, the frozen skin tissue was prepared using an Ambion RNA isolation Kit (Ambion Ltd. Huntingdon, Cambridgeshire, UK) according to the manufacturer’s instructions. RNA was measured using a NanoDrop ND-2000 spectrophotometer (Thermo Fisher Scientific, Waltham, MA, USA) after reverse transcription with oligo-dT primers using a PrimeScriptTM 1st strand cDNA synthesis Kit (Takara Bio Inc., Otsu, Japan), Real-time quantitative RT-PCR was conducted using IQ SYBR (Bio-Rad Laboratories, California, CA, USA) and 2 µL of cDNA in triplicate with glyceraldehyde 3-phosphate dehydrogenase (GAPDH) as a control. Before PCR amplification, the primers were denatured at 95 °C for 3 min. Amplification was repeated for 44 cycles at 95 °C for 10 s, 60 °C for 30 s, and 72 °C for 30 s. The PCR products were examined using a CFX Connect™ Real-Time PCR Detection System (Bio-Rad Laboratories). cDNA was probed with the following primers: IL-4 forward (5′-TCA TCG GCA TTT TGA ACG AG-3′); IL-4 reverse (5′-CGT TTG GCA CAT CCA TCT CC-3′); IFN-γ reverse (5′-GCC GTG GCA GTA ACA GCC-3′); GAPDH forward (5′-CAA GGA GTA AGA AAC CCT GGA CC-3′); GAPDH reverse (5′-GCC ATG GTA TTT GGA GCA CT-3′).

### 4.8. Assessment of Transepidermal Water Loss (TEWL) and Corneometer Units

TEWL measures the volume of water that crosses from inside an animal’s body to the surrounding atmosphere through the epidermal layer (skin) via diffusion and evaporation, and was evaluated on the final day of the experiment. TEWL in mouse dorsal skin was measured at a specific temperature (21–22 °C) and humidity (50%–55%), using a skin evaporative water recorder Tewameter TM300 (Courage and Khazaka, Cologne, Germany).

### 4.9. Statistical Analysis

Statistical analysis was performed with SPSS Statistics Software (SPSS Inc., Chicago, IL, USA). The data are presented as means ± standard error of the mean (SEM). Student’s *t*-test or one-way analysis of variance (ANOVA) followed by Tukey’s honestly significant difference (HSD) test were used to identify differences between multiple groups. Differences were considered significant at *p* < 0.05.

## References

[1] Leung D.Y. (2000). Atopic dermatitis: New insights and opportunities for therapeutic intervention. J. Allergy Clin. Immunol..

[2] Flohr C., Mann J. (2014). New insights into the epidemiology of childhood atopic dermatitis. Allergy.

[3] Totri C.R., Diaz L., Eichenfield L.F. (2014). 2014 update on atopic dermatitis in children. Curr. Opin. Pediatr..

[4] Spergel J.M., Paller A.S. (2003). Atopic dermatitis and the atopic march. J. Allergy Clin. Immunol..

[5] Von Kobyletzki L.B., Bornehag C.G., Hasselgren M., Larsson M., Lindstrom C.B., Svensson A. (2012). Eczema in early childhood is strongly associated with the development of asthma and rhinitis in a prospective cohort. BMC Dermatol..

[6] Bieber T. (2008). Atopic dermatitis. N. Engl. J. Med..

[7] Shimada Y., Takehara K., Sato S. (2004). Both Th2 and Th1 chemokines (TARC/CCL17, MDC/CCL22, and Mig/CXCL9) are elevated in sera from patients with atopic dermatitis. J. Dermatol. Sci..

[8] Hammad H., Charbonnier A.S., Duez C., Jacquet A., Stewart G.A., Tonnel A.B., Pestel J. (2001). Th2 polarization by Der p 1—Pulsed monocyte-derived dendritic cells is due to the allergic status of the donors. Blood.

[9] Hammad H., Smits H.H., Ratajczak C., Nithiananthan A., Wierenga E.A., Stewart G.A., Jacquet A., Tonnel A.B., Pestel J. (2003). Monocyte-derived dendritic cells exposed to Der p 1 allergen enhance the recruitment of Th2 cells: Major involvement of the chemokines TARC/CCL17 and MDC/CCL22. Eur. Cytokine Netw.

[10] Soumelis V., Reche P.A., Kanzler H., Yuan W., Edward G., Homey B., Gilliet M., Ho S., Antonenko S., Lauerma A. (2002). Human epithelial cells trigger dendritic cell mediated allergic inflammation by producing TSLP. Nat. Immunol..

[11] Ong P.Y., Leung D.Y. (2006). Immune dysregulation in atopic dermatitis. Curr. Allergy Asthma. Rep..

[12] Kim J.R., Choi J., Kim J., Kim H., Kang H., Kim E.H., Chang J.H., Kim Y.E., Choi Y.J., Lee K.W. (2014). 20-O-β-d-glucopyranosyl-20(*S*)-protopanaxadiol-fortified ginseng extract attenuates the development of atopic dermatitis-like symptoms in NC/Nga mice. J. Ethnopharmacol..

[13] Kim H., Kim J.R., Kang H., Choi J., Yang H., Lee P., Kim J., Lee K.W. (2014). 7,8,4ʹ-Trihydroxyisoflavone attenuates DNCB-induced atopic dermatitis-like symptoms in NC/Nga mice. PLoS ONE.

[14] Furue M., Terao H., Rikihisa W., Urabe K., Kinukawa N., Nose Y., Koga T. (2003). Clinical dose and adverse effects of topical steroids in daily management of atopic dermatitis. Br. J. Dermatol..

[15] Hengge U.R., Ruzicka T., Schwartz R.A., Cork M.J. (2006). Adverse effects of topical glucocorticosteroids. J. Am. Acad. Dermatol..

[16] Borowitzka M.A. (1986). Micro-algae as sources of fine chemicals. Microbiol. Sci..

[17] Morimura Y., Tamiya N. (1954). Preliminary experiments in the use of Chlorella as human food. Food Technol..

[18] Konishi F., Tanaka K., Himeno K., Taniguchi K., Nomoto K. (1985). Antitumor effect induced by a hot water extract of *Chlorella vulgaris* (CE): Resistance to meth-A tumor growth mediated by CE-induced polymorphonuclear leukocytes. Cancer Immunol. Immunother..

[19] Chovančíková M., Šimek V. (2001). Effects of hight-fat and *Chlorella vulgaris* feeding on changes in lipid metabolism in mice. Biol. Bratisl..

[20] Jong-Yuh C., Mei-Fen S. (2005). Potential hypoglycemic effects of Chlorella in streptozotocin-induced diabetic mice. Life Sci..

[21] Spolaore P., Joannis-Cassan C., Duran E., Isambert A. (2006). Commercial applications of microalgae. J. Biosci. Bioeng..

[22] Li L., Li W., Kim Y.H., Lee Y.W. (2013). *Chlorella vulgaris* extract ameliorates carbon tetrachloride-induced acute hepatic injury in mice. Exp. Toxicol. Pathol..

[23] Hasegawa T., Ito K., Ueno S., Kumamoto S., Ando Y., Yamada A., Nomoto K., Yasunobu Y. (1999). Oral administration of hot water extracts of *Chlorella vulgaris* reduces IgE production against milk casein in mice. Int. J. Immunopharmacol..

[24] Bae M.J., Shin H.S., Chai O.H., Han J.G., Shon D.H. (2013). Inhibitory effect of unicellular green algae (*Chlorella vulgaris*) water extract on allergic immune response. J. Sci. Food Agric..

[25] Spoehr H.A. (1951). Chlorella as a source of food. Proc. Am. Philos. Soc..

[26] Kwak J.H., Baek S.H., Woo Y., Han J.K., Kim B.G., Kim O.Y., Lee J.H. (2012). Beneficial immunostimulatory effect of short-term Chlorella supplementation: Enhancement of natural killer cell activity and early inflammatory response (randomized, double-blinded, placebo-controlled trial). Nutr. J..

[27] Palmer C.N., Irvine A.D., Terron-Kwiatkowski A., Zhao Y., Liao H., Lee S.P., Goudie D.R., Sandilands A., Campbell L.E., Smith F.J. (2006). Common loss-of-function variants of the epidermal barrier protein filaggrin are a major predisposing factor for atopic dermatitis. Nat. Genet..

[28] Werner Y., Lindberg M. (1985). Transepidermal water loss in dry and clinically normal skin in patients with atopic dermatitis. Acta Derm. Venereol..

[29] Leung D.Y., Boguniewicz M., Howell M.D., Nomura I., Hamid Q.A. (2004). New insights into atopic dermatitis. J. Clin. Investig..

[30] Lebman D.A., Coffman R.L. (1988). Interleukin 4 causes isotype switching to IgE in T cell-stimulated clonal B cell cultures. J. Exp. Med..

[31] Jujo K., Renz H., Abe J., Gelfand E.W., Leung D.Y. (1992). Decreased interferon gamma and increased interleukin-4 production in atopic dermatitis promotes IgE synthesis. J. Allergy Clin. Immunol..

